# Changes in Subclinical Biomarkers of Myocardial Injury and Inflammation and SARS-COV-2 Serological Analyses in a Cohort of Community-dwelling Adults

**DOI:** 10.21203/rs.3.rs-10298287/v1

**Published:** 2026-07-29

**Authors:** Meng Yuan, Elohor Oborevwori, Kayan Mehta, Raymond P. Owusu-Ansah, Tanish Madan, Deandra Thomas Romain, Gbolahan Olatunji, Shanshan Song, Melissa Hladek, Lori Sokoll, Phaedre Mohr, Jacky M Jennings, Shruti Mehta, Gregory D. Kirk, Cheryl R. Dennison Himmelfarb, Patricia M. Davidson, Wendy S. Post, Kelly Lowensen, Jason E. Farley, Yvonne Commodore-Mensah, Oluwabunmi Ogungbe

**Affiliations:** Johns Hopkins Bloomberg School of Public Health; Johns Hopkins University School of Nursing; Johns Hopkins University; Johns Hopkins Bloomberg School of Public Health; Johns Hopkins University; Scarborough General Hospital; Montefiore st Luke Cornwall; Johns Hopkins University School of Nursing; Johns Hopkins Bloomberg School of Public Health; Johns Hopkins Medicine; Johns Hopkins Hospital; Johns Hopkins Bloomberg School of Public Health; Johns Hopkins Bloomberg School of Public Health; Johns Hopkins Medicine; Johns Hopkins Medicine; Johns Hopkins University School of Nursing; Johns Hopkins Bloomberg School of Public Health; Johns Hopkins University School of Nursing; Johns Hopkins University School of Nursing; Johns Hopkins University School of Nursing; Johns Hopkins University School of Nursing

**Keywords:** SARS-CoV-2, Post-acute COVID-19 syndrome, Cardiac biomarkers, Subclinical cardiovascular disease, Health disparities

## Abstract

COVID-19 has been linked to heart-related complications in patients sick enough to be hospitalized, largely due to the strain severe illness places on the cardiovascular system. Whether milder, community-acquired infections carry similar risks is far less understood, and this question matters given how many people experienced mild or even symptom-free infections during the pandemic. We examined this question using blood samples from 607 community-dwelling adults, checking for antibodies that indicate past SARS-CoV-2 infection and tracking four biomarkers related to heart stress and inflammation over six months. About 15% of participants showed evidence of prior infection at the study's start, and this group tended to have higher body weight and included more Black or African American participants. Over the following six months, people with evidence of prior infection showed a larger rise in NT-proBNP, a protein that increases when the heart is under strain, compared to those without evidence of infection. Other markers showed less consistent patterns. These findings suggest that even mild, non-hospitalized COVID-19 may place subtle, lasting stress on the heart, particularly in people who already carry cardiovascular risk factors such as obesity or existing heart disease. The results support continued attention to heart health, vaccination, and monitoring after COVID-19 infection, especially in higher-risk communities.

## Introduction

The global COVID-19 pandemic brought renewed attention to the cardiovascular complications of acute viral infections. Early studies of hospitalized COVID-19 patients consistently reported elevated inflammatory markers such as interleukin-6 (IL-6) and C-reactive protein (CRP), alongside cardiac-specific biomarkers including high-sensitivity cardiac troponin I (hs-cTnI), N-terminal pro-B-type natriuretic peptide (NT-proBNP), and Galectin-3 (Gal-3).^1-4^ These biomarkers were selected because they capture distinct but complementary pathophysiological pathways: hs-cTnI reflects direct myocardial injury; NT-proBNP indicates myocardial wall stress and ventricular dysfunction; Gal-3 is a mediator of cardiac fibrosis and inflammation, activating cytokines such as IL-6 and TNF-α; and CRP serves as a systemic marker of acute phase inflammatory response. Cardiac troponin elevations, observed in 8% to 71% of hospitalized patients, were strongly associated with adverse outcomes.^2^ Gal-3, which activates inflammatory cytokines such as IL-6 and TNF-α, and NT-proBNP, is correlated with disease severity and mortality in these acute populations. ^2,3^ However, most research has focused on severe, hospitalized cases,^4^ and it remains unclear whether subclinical myocardial injury and persistent inflammation occur in non-hospitalized individuals with mild or asymptomatic SARS-CoV-2 infections, potentially increasing their long-term cardiovascular risk.^5-9^

Interpreting biomarker changes in community populations requires careful consideration of underlying cardiometabolic burden. Conditions including hypertension, diabetes, cardiovascular disease, chronic kidney disease, chronic lung disease, immunocompromised state, and cancer are independently associated with elevations in cardiac and inflammatory biomarkers, and their prevalence in urban populations may substantially shape biomarker profiles irrespective of SARS-CoV-2 exposure. Accounting for these factors is therefore essential to isolating the contribution of infection from background cardiometabolic risk.

The present study evaluated data from the C-FORWARD study^9^ to assess cardiac and inflammatory biomarkers in a community-based cohort, comparing six-month change in levels of hs-cTnI, NT-proBNP, Gal-3, and CRP across community-dwelling adults by their serological SARS-CoV-2 status (SARS-CoV-2 IgG, SARS-CoV-2 IgG II, SARS-CoV-2 IgM). Serostatus was defined using nucleocapsid IgG antibodies, which reflect prior natural infection, as nucleocapsid antibodies are not induced by spike-based vaccines and thus provide a more specific indicator of infection history independent of vaccination status. Additional serological measures are described in the Methods. By doing so, we aimed to better understand the intermediate to long-term F of SARS-CoV-2 infection on subclinical cardiovascular disease in community-dwelling persons.

## Results

### Baseline Characteristics of Study Participants by IgG defined COVID 19 status

Baseline characteristics of the 607 participants by SARS-CoV-2 nucleocapsid IgG-defined COVID-19 status are shown in Table 1. Of the 607 participants, 91 (15.0%) were IgG positive and 515 were IgG negative; one additional participant was excluded from stratified comparisons because of a quantity-not-sufficient IgG result. Compared with IgG-negative participants, IgG-positive participants were more likely to be Black or African American and less likely to be White (p = 0.002), and they had higher mean BMI (30.5 vs 28.6 kg/m^2^, p = 0.041) and obesity prevalence (49.5% vs 31.1%, p = 0.001). Other baseline characteristics were similar between groups (Table 1). To further characterize the cohort, we examined baseline characteristics stratified by IgG II antibody status **(Table S1)** and IgM antibody status **(Table S2)**. The distribution of cardiac and inflammatory biomarker levels among study participants is shown in Figure S1.

### Changes in Biomarkers Between Baseline and the 6-Month Visit by IgG defined COVID 19 status

Of 607 baseline participants, 312 (51%) had biomarker measurements at both baseline and 6 months and were included in paired analyses. Overall, within group changes from baseline to 6 months were not statistically significant for hs cTnI, NT proBNP, or CRP in either IgG group (Table 2). The only significant change was an increase in Galectin 3 among IgG positive participants, from 14.42 to 15.70 ng/mL (absolute change 1.28 ng/mL, 8.85% increase, p = 0.034), while Galectin 3 remained unchanged among IgG negative participants (15.26 to 15.28 ng/mL, p = 0.936) **(Table 2).**

**Figure 2:**
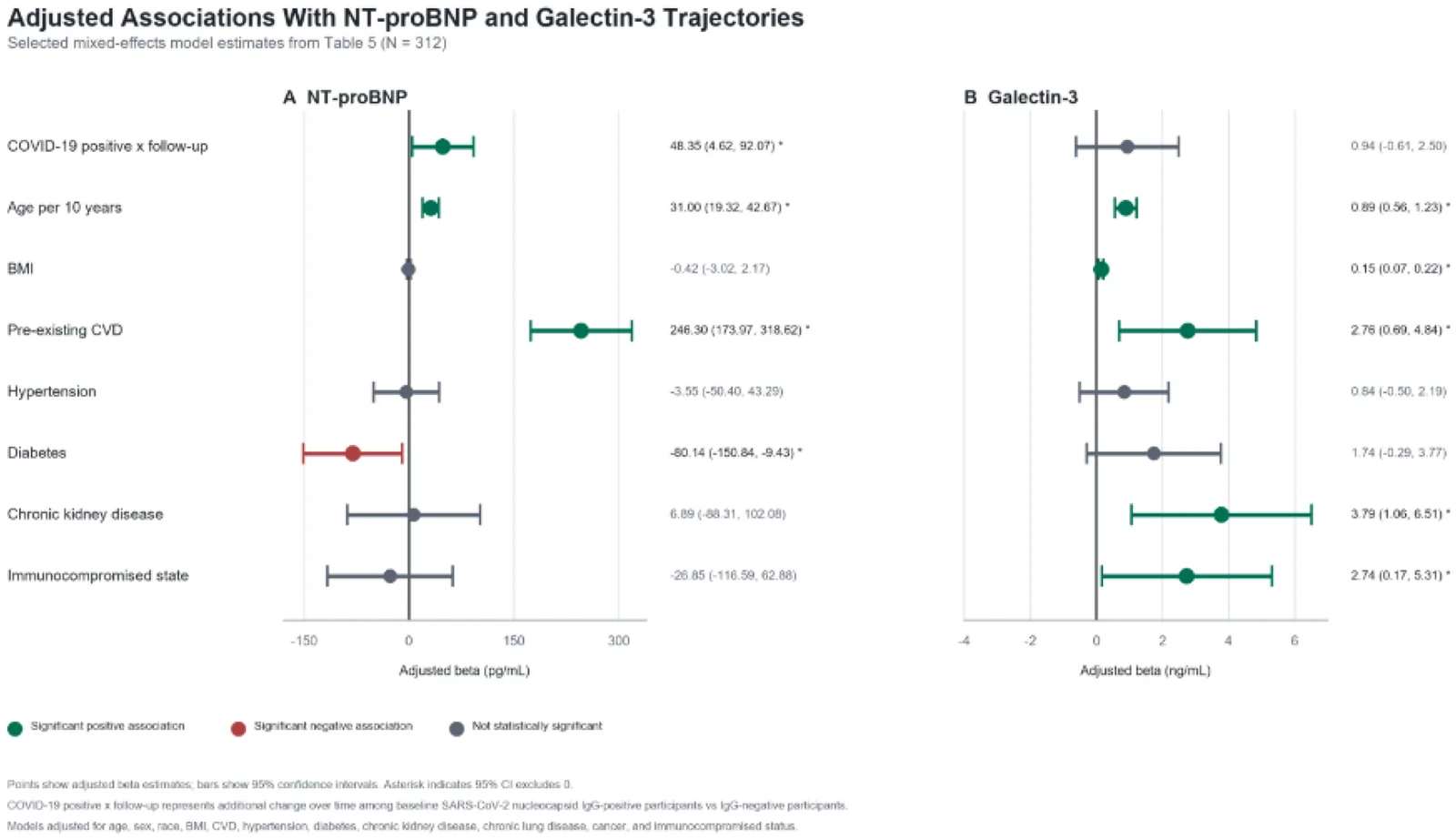
Adjusted Associations with NT-proBNP and Galectin 3-Trajectories

Figure 2 is consistent with Table 2, showing the clearest upward shift over time for Galectin 3 in IgG positive participants (n = 85) and overlapping confidence intervals across time for the other biomarkers within each IgG group (IgG negative n = 227). Related overall biomarker means changes and trajectory figures are provided in Figures S2 and S3.

Line plots showing mean biomarker levels with 95% confidence intervals from baseline to 6-month follow-up, stratified by baseline SARS-CoV-2 nucleocapsid IgG antibody status. **A**, hs-cTnI levels (ng/mL). **B**, NT-proBNP levels (pg/mL). **C**, Galectin-3 levels (ng/mL). **D**, CRP levels (mg/L). Blue lines show COVID-19-positive participants (IgG ≥ 1.4, n = 85); red lines show COVID-19-negative participants (n = 227). Error bars are 95% confidence intervals.

#### Linear Associations Between SARS-CoV-2 IgG-Defined COVID-19 Status, CVD Risk Factors, and Biomarker Changes

In Table 3, baseline IgG defined COVID 19 positivity was associated with a greater increase in NT proBNP from baseline to follow up. This association was present in the unadjusted model (β 43.99, 95% CI 3.52 to 84.45, p = 0.033) and remained significant after multivariable adjustment (β 54.21, 95% CI 9.69 to 98.72, p = 0.017). No significant associations were observed between COVID 19 status and changes in hs cTnI, Galectin 3, or CRP. In the adjusted model, diabetes and immunocompromised status were each associated with lower hs cTnI change, while Black race and higher BMI were associated with lower and higher CRP change, respectively (Table 3). All other estimates were not statistically significant and are shown in Table 3.

#### Odds of Elevated Biomarkers at Follow-up by Baseline Nucleocapsid IgG Positivity and Nucleocapsid IgG Positivity at Either Baseline or 6-Month Follow-up

Tables 4 and 4.1 presents the association between SARS-CoV-2 nucleocapsid IgG serostatus and odds of elevated cardiac and inflammatory biomarkers at follow-up under two exposure definitions. Using baseline IgG seropositivity as the exposure, there were no statistically significant associations with elevated hs-cTnI, NT-proBNP, Galectin-3, or CRP across unadjusted and adjusted models (Table 4). In a sensitivity analysis redefining exposure as IgG Positivity at either baseline or 6-Month follow-up, seropositivity was associated with higher odds of elevated NT-proBNP at follow-up in the more adjusted models (Model 2 OR 2.64, 95% CI 1.16 to 5.98; Model 3 OR 2.87, 95% CI 1.17 to 7.06), while associations for hs-cTnI, Galectin-3, and CRP remained non-significant (Table 4.1). Supplementary analyses of incident biomarker elevation mutually adjusted logistic regression models, and Cox proportional hazards models are presented in supplementary Tables S3–S5.

### Mixed-Effects Models of Biomarker Trajectories by SARS-CoV-2 IgG Status

In adjusted mixed-effects models, NT-proBNP increased more over follow-up among COVID-19-positive participants than among COVID-19-negative participants (COVID-19 × follow-up β = 48.35 pg/mL; 95% CI 4.62 to 92.07), indicating a greater rise in NT-proBNP over time in the IgG-positive group.In the same models, older age was associated with higher NT-proBNP (β = 31.00 pg/mL per 10-year increase; 95% CI 19.32 to 42.67) and higher Galectin-3 (β = 0.89 ng/mL per 10-year increase; 95% CI 0.56 to 1.23). Pre-existing cardiovascular disease was associated with higher hs-cTnI (β = 11.94 ng/L; 95% CI 3.49 to 20.40), higher NT-proBNP (β = 246.30 pg/mL; 95% CI 173.97 to 318.62), and higher Galectin-3 (β = 2.76 ng/mL; 95% CI 0.69 to 4.84). Higher BMI was associated with higher Galectin-3 (β = 0.15 ng/mL per kg/m^2^; 95% CI 0.07 to 0.22) and higher CRP (β = 0.38 mg/L per kg/m^2^; 95% CI 0.31 to 0.45). Black race, compared with White race, was associated with lower NT-proBNP (β = −45.40 pg/mL; 95% CI − 88.22 to − 2.59). Other covariates were not significantly associated with biomarker trajectories (Table 5).

## Discussion

This study evaluated longitudinal changes in subclinical biomarkers of myocardial injury and inflammation among community-dwelling adults in the C-FORWARD cohort. SARS-CoV-2 seropositivity was associated with elevated NT-proBNP in adjusted time-to-event analyses and with a modest increase in Galectin-3 over six months, while overall biomarker trajectories did not differ significantly by COVID-19 status. In contrast, pre-existing cardiovascular disease, hypertension, and elevated BMI were consistently and strongly associated with biomarker elevations, often exceeding the effects attributable to SARS-CoV-2 infection. Together, these findings suggest that cardiometabolic risk burden may be a dominant driver of subclinical cardiac stress in community populations, independent of SARS-CoV-2 exposure.

Our finding of elevated NT-proBNP among COVID-19-positive participants aligns with prior studies linking SARS-CoV-2 infection to myocardial stress and adverse cardiac outcomes, primarily in hospitalized populations.^14-15^ Evidence of persistent NT-proBNP elevation following infection also supports the possibility of ongoing cardiovascular effects beyond acute illness.^16^ These results extend prior work by demonstrating similar, though more modest, associations in a community-based cohort that includes individuals with mild or asymptomatic infection, a population largely absent from earlier biomarker studies, where the NT-proBNP signal was present but modest and emerged alongside strong competing associations with baseline cardiometabolic risk.

The observed increase in Galectin-3 among COVID-19-positive participants is consistent with its established role in inflammation and cardiac fibrosis in COVID-19.^17-19^ Prior studies have linked elevated Galectin-3 to worse clinical outcomes in hospitalized patients, and our findings suggest that smaller but detectable elevations may also occur in milder infections, though the clinical significance of such modest changes in a community setting warrants further investigation.

In contrast, hs-cTnI levels were not significantly associated with SARS-CoV-2 seropositivity after adjustment, consistent with evidence that troponin elevations are more pronounced in severe disease and hospitalized cohorts.^20-23^ This supports prior observations that troponin elevations in non-hospitalized populations often reflect non-ischemic myocardial stress rather than acute myocardial injury.^24^

A notable feature of our cohort was the demographic and clinical profile of COVID-19-positive participants, who were predominantly Black American women with higher rates of obesity and cardiovascular comorbidities. These patterns reflect well-documented disparities in COVID-19 burden and cardiovascular risk linked to structural and social determinants of health.^25-27^ This profile further reinforces the importance of accounting for cardiometabolic context when studying COVID-19-associated cardiac effects, as populations bearing the greatest infection burden often also carry the highest baseline cardiovascular risk.

A central finding of this study is the relative magnitude of cardiometabolic risk factors compared to SARS-CoV-2 serostatus as predictors of biomarker elevation. Baseline cardiovascular disease, hypertension, obesity, and diabetes were strong and consistent predictors of NT-proBNP elevation independent of infection status, and analyses additionally accounted for chronic kidney disease, chronic lung disease, immunocompromised state, and cancer. This pattern has meaningful implications for interpreting post-COVID biomarker research more broadly: in community cohorts where cardiometabolic burden is high, disentangling the contribution of SARS-CoV-2 exposure from underlying risk factor profiles is methodologically challenging and essential for accurate inference. Our study, by directly comparing these two sources of variation within the same analytic framework and adjusting for a comprehensive set of comorbidities, provides a more complete picture of what drives subclinical cardiac stress in urban populations.

The clinical and public health implications of these findings are twofold. First, the association between COVID-19 seropositivity and elevated NT-proBNP, along with increases in Galectin-3, supports consideration of post-COVID cardiovascular surveillance, particularly among individuals with pre-existing risk factors. Monitoring biomarkers of myocardial stress and inflammation may help identify individuals at increased risk for adverse outcomes and guide early preventive strategies.^28^ Second, the dominant influence of baseline cardiometabolic risk underscores the importance of comprehensive cardiovascular prevention efforts, including management of hypertension, obesity, and chronic disease, as well as addressing social determinants of health to reduce overall cardiovascular burden in urban populations. These findings also reinforce the importance of prioritizing COVID-19 vaccination among individuals with elevated cardiovascular risk, as preventing infection may help mitigate downstream cardiac stress and inflammatory effects.

This study has several important strengths, including a population-based, stratified sampling design that enhances relevance to urban populations, standardized measurement of validated cardiac and inflammatory biomarkers, and a longitudinal design with repeated measurements that enabled assessment of temporal biomarker changes.

Several limitations should be acknowledged. The proportion of SARS-CoV-2–IgG positive participants was relatively small (15%), restricting generalizability beyond similar urban populations. Reliance on serological testing limited precise characterization of infection timing, severity, and progression, including whether cases represented primary or repeat infections. Vaccination status could not be formally analyzed, as data collection occurred during a period when rollout was not yet fully implemented, resulting in incomplete records. Biomarker measurements were available at only two timepoints, and the six-month follow-up may not capture longer-term cardiovascular outcomes. Finally, the sample did not fully reflect Baltimore's demographics, with underrepresentation of African American residents.

## Conclusion

In community-dwelling adults, SARS-CoV-2 infection was associated with modest increases in NT-proBNP and Galectin-3 over six months, suggesting potential ongoing myocardial stress and low-grade inflammation even in mild or asymptomatic cases. These findings indicate that COVID-19 may contribute to subclinical cardiovascular risk beyond the acute illness and highlight the potential value of monitoring cardiac biomarkers to identify individuals at higher risk, guide early preventive strategies, and inform clinical management to mitigate long-term cardiovascular complications.

## Methods

### Design and Population

This cohort analysis used baseline and follow-up data from 607 community-dwelling adults participating in the Community Collaboration to Combat Coronavirus (C-FORWARD) study,^7^ a randomized controlled trial evaluating strategies for SARS-CoV-2 testing in Baltimore City. Between February 2021 and December 2022, 1,083 individuals were enrolled from English- and Spanish-speaking households using a multi-stage, population-representative, stratified sampling design with oversampling of historically harder-to-reach populations (e.g., Black, Latino/Latina, and low-income White residents).

Our current analysis includes participants with available blood samples and serological test results. All participants provided written informed consent for biorepository participation and laboratory analyses. The study protocol and substudy were approved by the Johns Hopkins Institutional Review Board (IRB00293365) and has clinical trial registration (NCT04673292).^7,8^ All methods were performed in accordance with the relevant guidelines and regulations.

### SARS-CoV-2 Serological Assessment

SARS-CoV-2 infection history was defined using serology. The primary exposure was baseline SARS-CoV-2 nucleocapsid IgG positivity, used as evidence of prior infection at study entry. Nucleocapsid IgG was measured in stored EDTA plasma and classified as positive at an index value of ≥ 1.4 (S/C). In secondary analyses, we also evaluated positive nucleocapsid IgG results at either baseline or the 6-month follow-up.

### Biomarker Assessment

Blood samples were collected at baseline and at the 6-month follow-up visit. The primary outcomes were subclinical myocardial inflammation and injury, assessed by four baseline biomarkers: hs-cTnI, NT-proBNP, Gal-3, and CRP. Biomarkers were dichotomized using established clinical thresholds: hs-cTnI ≥ 2.7 ng/L (limit of detection),^9^ NT-proBNP ≥ 125 pg/mL (heart failure screening threshold for age < 75 years),^10^ Gal-3 ≥ 18.5 ng/mL (cardiovascular risk stratification),^11^ and hs-CRP ≥ 0.9 mg/L (intermediate-to-high cardiovascular risk per AHA/CDC guidelines).^12^

### Clinical Laboratory Measurement

Additional baseline laboratory measurements included complete blood count (CBC) with differential and platelets, hepatic function measures, renal function measures (including creatinine and estimated glomerular filtration rate [eGFR] calculated using the Chronic Kidney Disease Epidemiology Collaboration [CKD-EPI] equation without race), and inflammatory markers, including lactate dehydrogenase (LDH), erythrocyte sedimentation rate (ESR), and interleukin-6 (IL-6).^13^ These measures were summarized as baseline characteristics and were not analyzed as outcomes.

### Covariates

Covariates were assessed at baseline via interviewer-administered questionnaires and standardized physical measurements, with follow-up ascertainment when needed. Sociodemographic covariates included age, sex, race/ethnicity, and body mass index (BMI; calculated from measured height and weight). Pre-existing conditions included hypertension, diabetes, cardiovascular disease, chronic kidney disease, chronic lung disease, immunocompromised state, and cancer.

### Statistical Analysis

We assessed missingness and outliers and winsorized continuous biomarker and laboratory variables at the 1st and 99th percentiles for descriptive analyses. Baseline characteristics were summarized for all 607 participants, while longitudinal analyses were limited to 312 participants with biomarker data at both baseline and 6-month follow-up. Group differences were assessed using chi-square or Fisher exact tests for categorical variables and t tests, analysis of variance, Wilcoxon rank-sum tests, or Kruskal-Wallis tests for continuous variables, as appropriate. Within-group biomarker changes were assessed using paired t tests. Linear regression models examined associations between baseline nucleocapsid IgG seropositivity and biomarker change.

Logistic regression models evaluated associations between SARS-CoV-2 serostatus and elevated biomarkers at follow-up, using baseline nucleocapsid IgG positivity as the primary exposure and IgG positivity at either baseline or follow-up as a secondary exposure. Three nested models were used: Model 1 adjusted for pre-existing cardiovascular disease; Model 2 additionally adjusted for age, sex, race, and BMI; and Model 3 further adjusted for hypertension, chronic lung disease, diabetes, chronic kidney disease, cancer, and immunocompromised status. Linear mixed-effects models examined repeated biomarker measurements from baseline to follow-up. Each model included time, baseline serostatus, and a time-by-serostatus interaction to assess whether biomarker trajectories differed by serostatus. Supplementary analyses examined alternative serologic markers, incident biomarker elevation, mutually adjusted logistic models, and time-to-event models, as described in supplementary document.

## Supplementary Material

This is a list of supplementary files associated with this preprint. Click to download.

- Supplementarydocument.docx
- Table2.docx

## Figures and Tables

**Figure 1:**
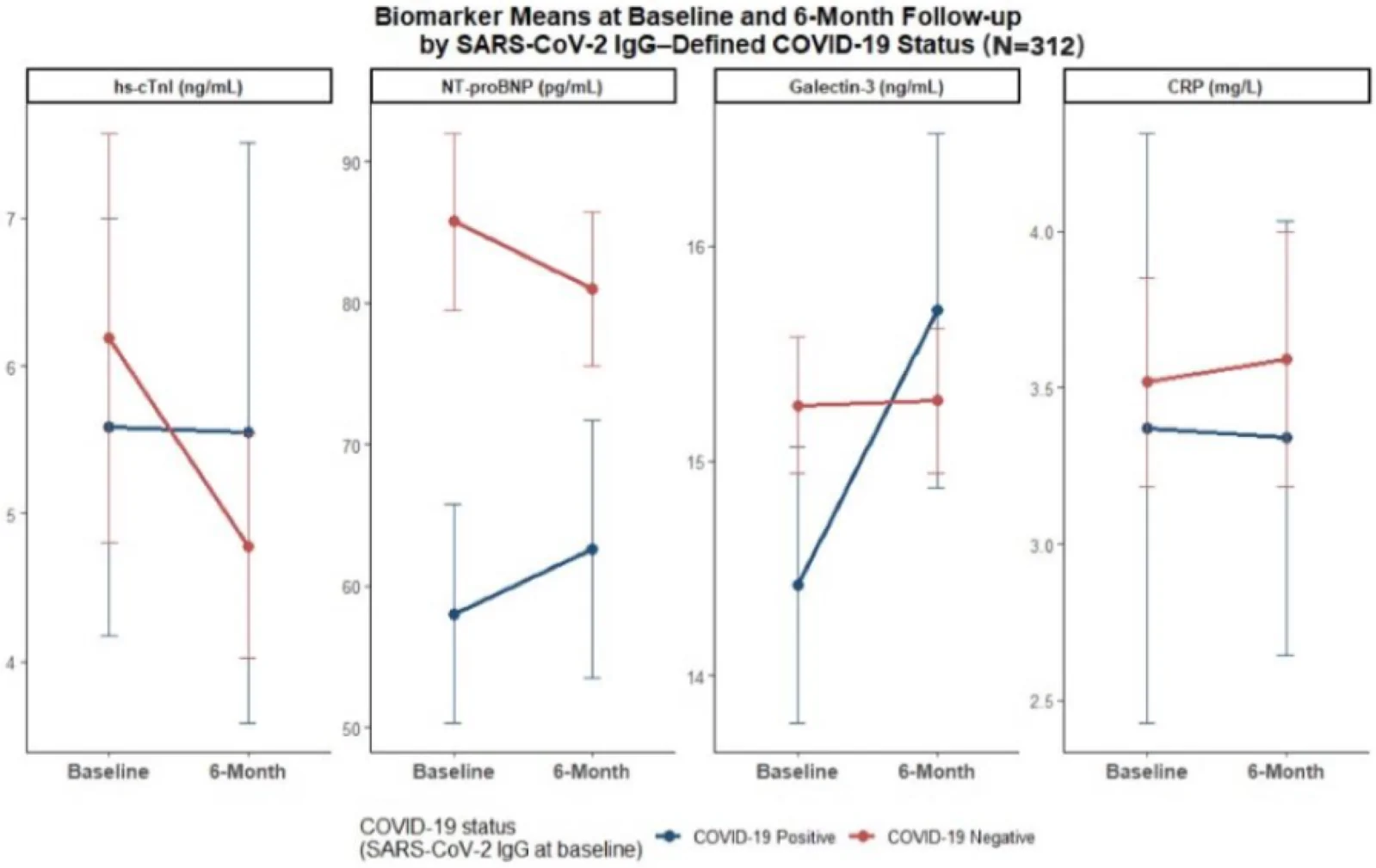
Biomarker Changes by SARS-CoV-2 IgG-Defined COVID-19 Status

**Table 1:** Baseline Characteristics of Study Participants (N = 607), Overall and by SARS-CoV-2 IgG–Defined COVID-19 Status

Characteristic	Total (N = 607)	COVID-19 Positive^a^ (n = 91)	COVID-19 Negative (n = 515)	p-value
Demographics				
Age, years (mean ± SD) ^b^	50.6 ± 16.8	51.0 ± 16.1	50.5 ± 16.9	0.809
**Sex at birth, n (%)**				1.000
Male	221 (36.4)	33 (36.3)	187 (36.3)	
Female	385 (63.4)	58 (63.7)	327 (63.5)	
**Race/Ethnicity, n (%)** ^c^				0.002*
Black or African American	240 (39.5)	50 (54.9)	190 (36.9)	
Asian	25 (4.1)	0 (0.0)	25 (4.9)	
White	333 (54.9)	39 (42.9)	293 (56.9)	
American Indian or Alaska Native	1 (0.2)	0 (0.0)	1 (0.2)	
Some other race	17 (2.8)	4 (4.4)	13 (2.5)	
Multiracial	15 (2.5)	2 (2.2)	13 (2.5)	
**Body mass index**				
BMI, kg/m^2^ (mean ± SD)	28.9 ± 7.7	30.5 ± 7.9	28.6 ± 7.7	0.041*
Obesity (BMI ≥ 30), n (%)	205 (33.8)	45 (49.5)	160 (31.1)	0.001*
**Pre-existing conditions, n (%)**				
Hypertension	186 (30.6)	24 (26.4)	162 (31.5)	0.407
Diabetes	71 (11.7)	16 (17.6)	55 (10.7)	0.086
Cardiovascular disease	38 (6.3)	7 (7.8)	31 (6.0)	0.778
Chronic kidney disease	19 (3.1)	2 (2.2)	17 (3.3)	0.752
Chronic lung disease	10 (1.6)	0 (0.0)	10 (1.9)	0.372
Immunocompromised state ^a^	26 (4.3)	3 (3.3%)	23 (4.5)	0.783
Cancer	13 (2.1)	1 (1.1)	12 (2.3)	0.703
**Cardiac & inflammatory biomarkers (median [IQR])** ^d^				
hs-cTnI, ng/L^e^	2.70 (2.70, 3.65)	2.70 (2.70, 3.15)	2.70 (2.70, 3.70)	0.086
NT-proBNP, pg/mL	47.60 (25.10, 93.10)	50.15 (23.70, 90.47)	47.45 (25.18, 94.10)	0.998
Galectin-3, ng/mL	14.30 (11.40, 17.90)	15.10 (11.60, 18.15)	14.10 (11.25, 17.90)	0.169
C-reactive protein, mg/L	1.58 (0.68, 4.33)	1.83 (0.74, 5.98)	1.58 (0.66, 4.33)	0.465
**Laboratory measurements (median [IQR])** ^f^				
Creatinine (0.76–1.27 mg/dL)	0.83 (0.71, 0.97)	0.81 (0.70, 1.08)	0.83 (0.72, 0.97)	0.982
eGFR (mL/min/1.73 m^2^)	90 (74, 105)	93 (82.3, 103.8)	89 (74, 105)	0.571
WBC (3.4–10.8 ×10^3^/L)	6.0 (5.0, 7.4)	5.6 (4.9, 6.5)	6.1 (5.1, 7.4)	0.064
RBC (3.77–5.28 ×10^6^/L)	4.65 (4.4, 5.02)	4.82 (4.5, 5.19)	4.63 (4.4, 4.97)	0.049 *
Hemoglobin (11.1–15.9 g/dL)	13.9 (13.0, 14.8)	13.9 (13.1, 15.4)	13.8 (13.0, 14.8)	0.473
Platelets (150–450 ×10^3^/L)	259 (218, 301)	260 (225, 290)	259 (215, 302)	0.902
Neutrophils (1.4–7.0 ×10^3^/L)	3.3 (2.6, 4.4)	3.1 (2.5, 3.7)	3.3 (2.6, 4.4)	0.090
Lymphocytes (0.7–3.1 ×10^3^/L)	1.9 (1.6, 2.3)	1.9 (1.6, 2.4)	1.9 (1.6, 2.3)	0.971
Monocytes (0.1–0.9 ×10^3^/L)	0.5 (0.4, 0.6)	0.5 (0.4, 0.5)	0.5 (0.4, 0.6)	0.143
Eosinophils (0.0–0.4 ×10^3^/L)	0.1 (0.1, 0.2)	0.1 (0.1, 0.2)	0.1 (0.1, 0.2)	0.137
Basophils (0.0–0.2 ×10^3^/L)	0.0 (0.0, 0.1)	0.0 (0.0, 0.1)	0.0 (0.0, 0.1)	0.087
AST (0–40 IU/L)	21 (17, 25)	20 (15.8, 24.0)	21 (17, 26)	0.114
ALT (0–44 IU/L)	17 (13, 24)	16 (13.8, 23.3)	17 (13.0, 24.8)	0.862
Alkaline phosphatase (39–117 IU/L)	78 (62, 97)	78.5 (65.8, 91.3)	78 (62, 97.3)	0.707
LDH (M: 118–273 IU/L)	215 (194, 245)	219 (204.5, 248.5)	215 (192, 244)	0.488
Albumin (3.5–5.5 g/dL)	4.5 (4.3, 4.8)	4.5 (4.3, 4.7)	4.5 (4.3, 4.8)	0.347

Note:

a COVID-19 infection was defined as positive or negative SARS-CoV-2 IgG antibody (detecting Nucleocapsid protein)

b Continuous variables are presented as mean ± standard deviation (SD) or median (interquartile range, IQR), as appropriate. Categorical variables are presented as n (%). p-values were obtained using Student’s t-test or Wilcoxon rank-sum test for continuous variables and χ^2^ or Fisher’s exact test for categorical variables.

c Race/ethnicity was collected as a multi-response variable; participants selecting ≥ 2 categories were classified as multiracial, and race percentages may exceed 100%.

d hs-cTnI, high sensitivity Troponin I; NT-proBNP, N-terminal pro-B-type natriuretic peptide; Gal-3, Galectin-3; eGFR, estimated glomerular filtration rate; WBC, White Blood Cell; RBC, Red Blood Cell; AST, Aspartate transaminase; ALT, Alanine transaminase; LDH, Lactate Dehydrogenase.

e For hs-cTnI, values reported below the assay’s lower limit of detection (e.g., “< 2.7 ng/L”) were imputed as the detection limit (2.7 ng/L).

f Reference ranges shown reflect general adult reference intervals; LDH, male reference range (118-273IU/L))

a Includes autoimmune conditions

* Statistically significant at p < 0.05

**Table 3:** Linear regression models of baseline IgG defined COVID 19 status and covariates on biomarker change from baseline to follow up

Exposure variables	hs cTnI change^a^ (95% CI) p value	NT proBNP change (95% CI) p value	Galectin 3 change (95% CI) p value	CRP change (95% CI) p value
Unadjusted models				
COVID 19 positive^b^	1.46 (−4.21, 7.14), p = 0.613	**43.99 (3.52, 84.45), p = 0.033**	1.25 (−0.29, 2.78), p = 0.110	−0.41 (−3.10, 2.28), p = 0.764
Age (per 10 year increase)	0.13 (−0.94, 1.21), p = 0.807	−0.62 (−8.34, 7.09), p = 0.874	0.24 (−0.04, 0.53), p = 0.097	−0.03 (−0.51, 0.45), p = 0.902
Female sex	−2.66 (−6.37, 1.06), p = 0.160	−3.21 (−29.97, 23.56), p = 0.814	0.70 (−0.31, 1.70), p = 0.174	0.80 (−0.86, 2.45), p = 0.343
Black race (vs White)	−0.35 (−4.05, 3.35), p = 0.852	2.30 (−24.28, 28.87), p = 0.865	−0.03 (−1.03, 0.97), p = 0.954	−0.88 (−2.51, 0.74), p = 0.286
BMI	−0.10 (−0.35, 0.15), p = 0.427	1.07 (−0.73, 2.87), p = 0.243	0.01 (−0.05, 0.08), p = 0.747	0.09 (−0.01, 0.20), p = 0.079
Pre-existing cardiovascular disease	−5.96 (−11.75, −0.16), p = 0.044	84.30 (33.90, 134.69), p = 0.001	−1.23 (−3.10, 0.65), p = 0.198	2.18 (−0.82, 5.18), p = 0.153
Hypertension	−2.15 (−5.43, 1.13), p = 0.198	2.57 (−26.40, 31.54), p = 0.861	−0.15 (−1.22, 0.91), p = 0.777	0.73 (−1.05, 2.52), p = 0.419
Diabetes	−6.18 (−11.09, −1.27), p = 0.014	5.05 (−38.84, 48.93), p = 0.821	0.18 (−1.42, 1.78), p = 0.826	0.17 (−2.53, 2.87), p = 0.904
Chronic kidney disease	1.14 (−6.76, 9.03), p = 0.777	31.11 (−38.28, 100.49), p = 0.378	0.58 (−1.95, 3.10), p = 0.654	1.68 (−2.38, 5.75), p = 0.415
Chronic lung disease	1.37 (−10.23, 12.97), p = 0.816	−28.29 (−130.32, 73.74), p = 0.586	3.39 (−0.31, 7.08), p = 0.072	−0.01 (−5.71, 5.69), p = 0.998
Immunocompromised state	−16.94 (−24.54, −9.33), p = 0.000	−41.32 (−110.73, 28.08), p = 0.242	1.92 (−0.63, 4.47), p = 0.139	0.04 (−3.84, 3.91), p = 0.986
Cancer	2.41 (−9.11, 13.92), p = 0.681	−10.84 (−112.68, 91.01), p = 0.834	−0.82 (−4.57, 2.92), p = 0.665	−0.74 (−6.40, 4.92), p = 0.797
**Adjusted model** ^c^				
COVID 19 positive	1.95 (−2.99, 6.89), p = 0.438	**54.21 (9.69, 98.72), p = 0.017**	0.84 (−0.78, 2.45), p = 0.309	−0.06 (−3.03, 2.91), p = 0.968
Age (per 10 year increase)	0.93 (−0.13, 1.99), p = 0.086	−3.81 (−13.40, 5.78), p = 0.434	0.20 (−0.14, 0.55), p = 0.253	0.03 (−0.56, 0.62), p = 0.926
Female sex	−2.14 (−5.50, 1.22), p = 0.210	0.59 (−29.68, 30.86), p = 0.969	0.52 (−0.57, 1.61), p = 0.350	1.18 (−0.68, 3.04), p = 0.213
Black race (vs White)	1.66 (−2.23, 5.55), p = 0.402	−20.89 (−55.96, 14.17), p = 0.242	−0.13 (−1.40, 1.14), p = 0.844	**−2.30 (−4.45, −0.16), p = 0.036**
BMI	0.13 (−0.10, 0.37), p = 0.271	1.09 (−1.04, 3.21), p = 0.315	0.02 (−0.06, 0.10), p = 0.606	0.14 (0.01, 0.26), p = 0.031
Pre-existing cardiovascular disease	−6.54 (−13.11, 0.03), p = 0.051	120.99 (61.76, 180.21), p = 0.000	−1.38 (−3.53, 0.77), p = 0.206	3.25 (−0.27, 6.76), p = 0.070
Hypertension	−3.41 (−7.67, 0.84), p = 0.116	−1.47 (−39.83, 36.89), p = 0.940	−0.68 (−2.07, 0.72), p = 0.340	0.71 (−1.57, 2.99), p = 0.541
Diabetes	**−7.08 (−13.50, −0.66), p = 0.031**	−23.45 (−81.36, 34.46), p = 0.426	0.55 (−1.55, 2.65), p = 0.607	−1.18 (−4.81, 2.44), p = 0.520
Chronic kidney disease	5.35 (−3.30, 14.00), p = 0.224	35.05 (−42.90, 113.00), p = 0.377	1.04 (−1.79, 3.87), p = 0.471	2.36 (−2.25, 6.96), p = 0.314
Chronic lung disease	7.42 (−4.91, 19.75), p = 0.237	−51.06 (−162.24, 60.12), p = 0.367	2.82 (−1.21, 6.85), p = 0.170	−2.58 (−8.75, 3.59), p = 0.410
Immunocompromised state	**−18.51 (−26.66, −10.36), p = 0.000**	−42.23 (−115.71, 31.25), p = 0.259	1.29 (−1.38, 3.95), p = 0.343	0.80 (−3.26, 4.87), p = 0.698
Cancer	3.79 (−7.80, 15.37), p = 0.520	−21.18 (−125.65, 83.30), p = 0.690	−0.99 (−4.78, 2.80), p = 0.609	−1.85 (−7.61, 3.91), p = 0.528

Note:

a Beta estimates represent the mean difference in biomarker change associated with the exposure (or per unit increase for continuous variables). 95% confidence intervals and two-sided p values are shown.

b COVID 19 positivity was defined by baseline SARS CoV 2 nucleocapsid IgG serostatus (IgG positive vs IgG negative).

c Adjusted model: One multivariable linear regression model that includes COVID 19 status and all covariates listed in the left column in the same model. Each estimate represents the association for that variable after accounting for the other listed covariates.

**Table 4:** Association Between Baseline SARS-CoV-2 Nucleocapsid IgG Seropositivity and Elevated Cardiac/Inflammatory Biomarkers at Follow-Up (N = 312)

Elevated cardiac and inflammatory biomarkers	Unadjusted OR (95% CI), p value	Model 1 * OR (95% CI), p value	Model 2‡ OR (95% CI), p value	Model 3§ OR (95% CI), p value
High sensitivity Troponin I (hs cTnI > 2.7 ng/L)	0.97 (0.47, 2.00), p = 0.936	1.06 (0.50, 2.25), p = 0.871	0.93 (0.41, 2.14), p = 0.872	1.05 (0.45, 2.48), p = 0.910
N terminal pro BNP (NT pro BNP ≥ 125 pg/mL)	1.18 (0.48, 2.87), p = 0.719	1.26 (0.49, 3.25), p = 0.629	1.64 (0.57, 4.70), p = 0.359	1.95 (0.63, 6.05), p = 0.250
Galectin 3 (≥ 18.5 ng/mL)	1.11 (0.47, 2.59), p = 0.811	1.13 (0.48, 2.68), p = 0.778	1.12 (0.45, 2.82), p = 0.808	1.00 (0.36, 2.77), p = 0.996
CRP (≥ 0.9 mg/L)	0.94 (0.44, 2.03), p = 0.878	0.98 (0.45, 2.12), p = 0.963	0.78 (0.31, 1.96), p = 0.599	0.74 (0.28, 1.96), p = 0.538

Bold, p < 0.05; 95% CI, 95% confidence interval.

* Model 1: adjusted for pre-existing cardiovascular disease.

‡ Model 2: Model 1 variables plus age, biological sex, race, and BMI.

§ Model 3: Model 2 variables plus hypertension, chronic lung disease, diabetes, chronic kidney disease, cancer, and immunocompromised (immune) conditions.

**Table 4.1:** Association Between IgG Positivity at Either Baseline or 6-Month Follow-up and Elevated Cardiac/Inflammatory Biomarkers at Follow-Up (N = 312)

Elevated cardiac and inflammatory biomarkers	Unadjusted OR (95% CI), p value	Model 1 * OR (95% CI), p value	Model 2‡ OR (95% CI), p value	Model 3§ OR (95% CI), p value
High sensitivity Troponin I (hs-cTnI > 2.7 ng/L)	0.70 (0.41, 1.20), p = 0.194	0.76 (0.43, 1.33), p = 0.333	0.77 (0.41, 1.44), p = 0.406	0.76 (0.39, 1.47), p = 0.415
N terminal pro BNP (NT-proBNP ≥ 125 pg/mL)	1.37 (0.72, 2.61), p = 0.335	1.66 (0.82, 3.35), p = 0.156	**2.64 (1.16, 5.98), p = 0.020**	**2.87 (1.17, 7.06), p = 0.022**
Galectin-3 (≥ 18.5 ng/mL)	0.91 (0.48, 1.71), p = 0.761	0.91 (0.47, 1.77), p = 0.776	1.04 (0.51, 2.13), p = 0.904	1.11 (0.51, 2.45), p = 0.787
CRP (≥ 0.9 mg/L)	1.03 (0.59, 1.79), p = 0.923	1.00 (0.57, 1.78), p = 0.990	0.99 (0.52, 1.91), p = 0.979	0.89 (0.44, 1.79), p = 0.745

Bold, p < 0.05; 95% CI, 95% confidence interval.

* Model 1: adjusted for pre-existing cardiovascular disease.

‡ Model 2: Model 1 variables plus age, biological sex, race, and BMI.

§ Model 3: Model 2 variables plus hypertension, chronic lung disease, diabetes, chronic kidney disease, cancer, and immunocompromised (immune) conditions.

**Table 5:** Mixed-Effects Regression Results for Biomarker Trajectories (N = 312)

Exposure Variables	hs-cTnI (ng/L) β^a^ (95% CI)	NT-proBNP (pg/mL) β (95% CI)	Galectin-3 (ng/mL) β (95% CI)	CRP (mg/L) β (95% CI)
Time (follow-up vs baseline; ref = COVID-negative)^b^	−1.00 (−2.75, 0.74)	−4.84 (−20.05, 10.37)	0.06 (−0.48, 0.60)	0.40 (−0.45, 1.24)
COVID-19 Positive (baseline IgG)	−0.30 (−7.13, 6.54)	20.65 (−37.93, 79.24)	−1.22 (−2.96, 0.52)	−0.24 (−2.44, 1.96)
COVID-19 Positive × Follow-up	1.08 (−3.94, 6.10)	**48.35 (4.62, 92.07)**	0.94 (−0.61, 2.50)	−0.35 (−2.94, 2.24)
Covariates				
Age (per 10 years)	−0.66 (−2.02, 0.71)	**31.00 (19.32, 42.67)**	**0.89 (0.56, 1.23)**	−0.19 (−0.53, 0.14)
Female sex	0.89 (−3.41, 5.20)	7.29 (−29.54, 44.12)	0.04 (−1.01, 1.09)	0.65 (−0.40, 1.70)
Black race (vs White)	−2.13 (−7.13, 2.88)	**−45.40 (−88.22, −2.59)**	−0.56 (−1.79, 0.67)	−0.43 (−1.67, 0.80)
BMI	−0.06 (−0.37, 0.24)	−0.42 (−3.02, 2.17)	**0.15 (0.07, 0.22)**	**0.38 (0.31, 0.45)**
Pre-existing CVD	**11.94 (3.49, 20.40)**	**246.30 (173.97, 318.62)**	**2.76 (0.69, 4.84)**	1.76 (−0.28, 3.80)
Hypertension	1.48 (−4.00, 6.96)	−3.55 (−50.40, 43.29)	0.84 (−0.50, 2.19)	0.26 (−1.07, 1.58)
Diabetes	1.67 (−6.60, 9.93)	**−80.14 (−150.84, −9.43)**	1.74 (−0.29, 3.77)	−0.35 (−2.38, 1.68)
Chronic kidney disease	−3.03 (−14.16, 8.09)	6.89 (−88.31, 102.08)	**3.79 (1.06, 6.51)**	−0.44 (−3.12, 2.24)
Chronic lung disease	−5.53 (−21.40, 10.34)	−38.00 (−173.78, 97.77)	0.11 (−3.78, 4.00)	−0.30 (−4.04, 3.44)
Immunocompromised state	9.58 (−0.91, 20.07)	−26.85 (−116.59, 62.88)	**2.74 (0.17, 5.31)**	0.37 (−2.10, 2.84)
Cancer	−1.45 (−16.37, 13.46)	−40.56 (−168.14, 87.03)	−2.40 (−6.05, 1.26)	0.91 (−2.60, 4.41)

Note:

a β (95% CI) from linear mixed-effects models for continuous biomarker levels measured at two time points (baseline and follow-up). Each row is a covariate, and each β represents the association with the biomarker after adjusting all other covariates listed.

b COVID-19 infection was defined as positive or negative SARS-CoV-2 IgG antibody (detecting Nucleocapsid protein) ; COVID-19 Positive (baseline IgG) represents the baseline difference in biomarker level between COVID-19–positive and COVID-19–negative participants; Time (follow-up vs baseline; ref = COVID-negative) represents the mean change from baseline to follow-up in the COVID-19–negative reference group, holding covariates constant. COVID-19 Positive × Follow-up is the interaction term and represents the additional change from baseline to follow-up in the COVID-19–positive group relative to the COVID-19–negative group (difference-in-differences).

## Data Availability

The data that support the findings of this study are available from the corresponding author upon reasonable request.
